# Indomethacin induces apoptosis via a MRP1-dependent mechanism in doxorubicin-resistant small-cell lung cancer cells overexpressing MRP1

**DOI:** 10.1038/sj.bjc.6604010

**Published:** 2007-10-16

**Authors:** D J A de Groot, M van der Deen, T K P Le, A Regeling, S de Jong, E G E de Vries

**Affiliations:** 1 Department of Medical Oncology, University of Groningen and University Medical Center, PO Box 30.001, 9700 RB, Groningen, The Netherlands

**Keywords:** indomethacin, apoptosis, MRP1, doxorubicin resistance, SCLC

## Abstract

Small-cell lung cancers (SCLCs) initially respond to chemotherapy, but are often resistant at recurrence. The non-steroidal anti-inflammatory drug indomethacin is an inhibitor of multidrug resistance protein 1 (MRP1) function. The doxorubicin-resistant MRP1-overexpressing human SCLC cell line GLC_4_-Adr was highly sensitive for indomethacin compared with the parental doxorubicin-sensitive line GLC_4_. The purpose of this study was to analyse the relationship between hypersensitivity to indomethacin and MRP1 overexpression. The experimental design involved analysis of the effect of MRP1 downregulation on indomethacin-induced cell survival and apoptosis in GLC_4_-Adr and GLC_4_, using siRNA. In addition the effect of indomethacin on glutathione levels and mitochondrial membrane potential was investigated. Small interfering RNAs directed against MRP1 reduced MRP1 mRNA levels twofold and reduced efflux pump function of MRP1, which was reflected by a 1.8-fold higher accumulation of MRP1 substrate carboxyfluorescein, in si-MRP1 versus si-Luciferase-transfected GLC_4_-Adr cells. Multidrug resistance protein 1 downregulation decreased initial high apoptosis levels 2-fold in GLC_4_-Adr after indomethacin treatment for 24 h, and increased cell survival (IC_50_) from 22.8±2.6 to 30.4±5.1 *μ*M following continuous indomethacin exposure. Multidrug resistance protein 1 downregulation had no effect on apoptosis in GLC_4_ or on glutathione levels in both lines. Although indomethacin (20 *μ*M) for 2 h decreased glutathione levels by 31.5% in GLC_4_-Adr, complete depletion of cellular glutathione by L-buthionine (S,R)-sulphoximine only resulted in a small increase in indomethacin-induced apoptosis in GLC_4_-Adr, demonstrating that a reduced cellular glutathione level is not the primary cause of indomethacin-induced apoptosis. Indomethacin exposure decreased mitochondrial membrane potential in GLC_4_-Adr cells, suggesting activation of the mitochondrial apoptosis pathway. Indomethacin induces apoptosis in a doxorubicin-resistant SCLC cell line through an MRP1-dependent mechanism. This may have implications for the treatment of patients with MRP1-overexpressing tumours.

Lung cancer is the tumour type with the highest incidence in males in the Western world, and its incidence in females is rising. Small-cell lung cancer (SCLC) represents about 25% of all lung cancers. Small-cell lung cancers are well known for their initial sensitivity to chemotherapeutic agents. However, they frequently recur, and it is at this time that the tumours become drug resistant (Glisson, 2003). Common mechanisms of drug resistance are the overexpression of drug transporters and the resistance to apoptosis induction in tumour cells. The ATP-binding cassette (ABC) family of transport proteins is the major family of drug transporters. The multidrug resistance protein 1 (MRP1), a member of the ABC family of drug transporters, can act as an efflux pump for a number of chemically unrelated agents. Intracellular glutathione (GSH) can be conjugated to these agents by glutathione-*S*-transferase. Multidrug resistance protein 1 transports GSH, GSH conjugates and unconjugated cytotoxic drugs to the extracellular compartment (Cole *et al*, 1994; Müller *et al*, 1994; Zaman *et al*, 1994; Paul *et al*, 1996; Ballatori *et al*, 2005). Glutathione is required for several other cellular functions such as protein and DNA synthesis, cell cycle regulation, protection against oxidative damage and detoxification of toxins (Wang and Ballatori, 1998).

The non-steroidal anti-inflammatory drug indomethacin is a well-known inhibitor of MRP1 function. It inhibits glutathione-*S*-transferase and also functions as a direct substrate for MRP1 (Draper *et al*, 1997; Touhey *et al*, 2002). In addition, in low concentrations indomethacin can increase GSH efflux (Evers *et al*, 2000). We previously reported that exposure of the MRP1-overexpressing doxorubicin-resistant SCLC cell line GLC_4_-Adr to indomethacin resulted in caspase-8- and caspase-9-dependent apoptosis induction, suggesting the involvement of the extrinsic and intrinsic apoptosis pathways. In contrast, exposure of the parental cell line GLC_4_ to indomethacin did not induce apoptosis (De Groot *et al*, 2005). Indomethacin as well as MK571 increase doxorubicin sensitivity in these GLC_4_ cell lines. The use of this isogenic model implies that factors rendering GLC_4_-Adr resistant to doxorubicin are very likely also be responsible for indomethacin sensitivity. Studies investigating the increase in drug sensitivity after indomethacin exposure in human and murine cell lines showed that the drug-sensitizing effect of indomethacin was not prostaglandin dependent, but was dependent on glutathione-*S*-transferase, or caused by direct inhibition of glutathione-*S*-transferase (Draper *et al*, 1997). Insight into this mechanism might result in a simple, relatively non-toxic way to exploit MRP1 overexpression for apoptosis induction in chemotherapy-resistant cells. In this study, we therefore investigated whether indomethacin-induced apoptosis is related to MRP1 overexpression. In addition the mechanism of indomethacin-induced apoptosis with respect to MRP1 function and GSH levels was studied.

## MATERIALS AND METHODS

### Cell lines

The GLC_4_ cell line was derived from a pleural effusion in our laboratory and kept in culture in RPMI 1640 medium supplemented with 10% heat-inactivated foetal calf serum (FCS) (both from Life Technologies, Breda, The Netherlands). The GLC_4_-Adr sub-line acquired resistance not only to doxorubicin but also to a wide range of other chemotherapeutic agents, by stepwise increases in doxorubicin concentrations in the culture medium (Zijlstra *et al*, 1987; De Jong *et al*, 1990; Meijer *et al*, 1991; Müller *et al*, 1994). GLC_4_-Adr is 190.6±16.2-fold more resistant to doxorubicin than GLC_4_. The doxorubicin resistance in GLC_4_-Adr is due to a downregulation of the activity of DNA-topoisomerase II (TOPO II) and amplification and consequent 79-fold overexpression of the *MRP1* gene. GLC_4_-Adr was maintained in culture medium 1.2 *μ*M doxorubicin twice weekly. GLC_4_-Adr was cultured without doxorubicin for 20 days before experiments. Cells were cultured at 37°C in a humidified atmosphere with 5% CO_2_. Cells from exponentially growing cultures were used for all experiments. Forty-eight hours of indomethacin (50 *μ*M) treatment induces 50.6±14.6% apoptosis in GLC_4_-Adr and 2.1±1.5% apoptosis in GLC_4_.

### Chemicals, media and reagents

HAM/F12 and DMEM medium (minimal essential medium (MEM, supplemented with Earle's salts and L-glutamine), oligofectamine, RPMI 1640, phosphate-buffered saline (PBS), Hoechst 33258, MitoTracker Red CM-H_2_XRos and Trizol were purchased from Invitrogen Life Technologies (Breda, The Netherlands)). Carboxyfluorescein diacetate (CFDA), 3-[4,5-dimethylthiazol-2-yl]-2,5-diphenyltetrazolium bromide (MTT), L-buthionine (S,R)-sulphoximine (BSO), glutathione reductase, nicotinamide adenine dinucleotide phosphate (NADPH), trichloric acid (TCA), GSH, 5,5′-dithio-bis(2-nitrobenzoic acid) (DTNB) and propidium iodide (PI) were obtained from Sigma-Aldrich BV (Zwijndrecht, The Netherlands); DNase-I was from Roche Diagnostics (Mannheim, Germany), doxorubicin-HCl from Pharmachemie BV (Haarlem, The Netherlands), ethylenedinitrilo tetraacetic acid disodiumsalt dihydrate (EDTA) from Merck (Darmstadt, Germany). MK571 was purchased from Omnilabo (Breda, The Netherlands), the qPCR core kit from Eurogentec (Seraing, Belgium), the RNeasy kit from Qiagen (Venlo, The Netherlands) and Vitrogen from Nutacon (Leimuiden, The Netherlands).

### Flow cytometric detection of MRP1 activity

To determine MRP1 activity, cells were incubated with 0.1 *μ*Mcarboxyfluorescein diacetate as described previously (Van der Kolk *et al*, 1998), with slight modifications. Carboxyfluorescein diacetate is converted intracellularly to carboxyfluorescein (CF), which is a fluorescent MRP1 substrate. To establish the effect of indomethacin on the activity of MRP1, 1 × 10^6^ cells were incubated in 0.5 ml RPMI 1640 medium (37°C, 5% CO_2_, 1 h) with CFDA. MK571 (50 *μ*M) served as a positive control for the inhibition of MRP1 activity. Cells were pelleted for 15 s at 12 000 **g** and resuspended in 350 *μ*l ice-cold RPMI medium with 0.1 *μ*g ml^−1^ PI to distinguish dead cells from living cells. Fluorescence of CF was analysed with a FACS Caliber flow cytometer (BD Biosciences, San Jose, CA, USA). A total of 10 000 events were measured per sample. The Winlist 5.1 programme (Verity Software House Inc., Topsham, ME, USA) was used to calculate mean fluorescence intensity (MFI) values. The efflux-blocking factor (BF) was defined as the ratio between MFI of substrate plus modulator and MFI of substrate. All measurements were corrected for the negative control (without indomethacin). Experiments were performed in triplicate.

### RNA interference

To explore the role of MRP1 in indomethacin-induced apoptosis, siRNAs directed against MRP1 (si-MRP1) and luciferase (si-Luciferase, negative control) were purchased from Eurogentec (Maastricht, The Netherlands). The sense sequence for si-MRP1 was 5′-GGAGUGGAACCCCUCUCUG-3′ and the antisense sequence was 5′-CAGAGAGGGGUUCCACUCC-3′. For si-Luciferase, the sense sequence was 5′-CUUACGCUGAGUACUUCGA-3′ and the antisense sequence was 5′-UCGAAGUACUCAGCGUAAG-3′.

GLC_4_ and GLC_4_-Adr cells were seeded in six-wells plates at a concentration of 3 × 10^5^ well^−1^. The next day, transfection was performed using 200 nM oligonucleotides with Oligofectamine and RPMI 1640, in the absence of FCS according to the manufacturer's instructions. After 4 h the medium containing FCS was added to an FCS solution of final concentration of 10%. Multidrug resistance protein 1 function assays with flow cytometre and RNA extraction were carried out 48 h after siRNA transfection.

### RNA isolation and quantitative RT–PCR

Cells treated with siRNA for 48 h were washed with ice-cold PBS and resuspended in 1 ml of Trizol. After 5 min incubation at room temperature, the lysate was stored at −80°C until use. RNA was isolated according to standard manufacturer's protocols, followed by a DNase-I treatment. For RNA purification, the RNeasy kit was used following standard procedures. RNA (800 ng) was subjected to a complementary DNA (cDNA) synthesis reaction.

Quantitative reverse transcriptase–polymerase chain reaction (RT–PCR) was performed using the ABI PRISM 7700 sequence detector (Applied Biosystems, Foster City, USA, USA) as described previously (Ros *et al*, 2003). In brief, a qPCR core kit was used and the PCR mixture contained 900 nM of sense and antisense primers, 200 nM of fluorogenic probe (labelled by a 5′ FAM reporter and a 3′ TAMRA quencher). Each sample was analysed in duplicate. Primer sequences for MRP1 were as follows: for the sense strand, 5′-GGTGGGCCGAGTGGAATT-3′; for the antisense strand, 5′-TTGATGTGCCTGAGAACGAAGT-3′ and for the probe strand, 5′FAM-CTGCCTGCGCTACCGAGAGGACCT-TAMRA3′. Primer sequences for the housekeeping gene GAPDH were for the sense strand 5′-GGTGGTCTCCTCTGACTTCAACA-3′, for the anti-sense strand 5′-GTGGTCGTTGAGGGCAATG-3′ and for the probe strand 5′FAM-ACACCCACTCCTCCACCTTTGACGC-TAMRA3′. Cycle numbers at which the sample fluorescence signal increases above a fixed threshold level (*C*_t_ value) correlate inversely with mRNA levels.

### MTT assay

The cell lines were cultured in HAM/F12 and DMEM medium (1 : 1) supplemented with 20% FCS. The effect of doxorubicin and indomethacin on survival was tested in an MTT assay as described previously (Timmer-Bosscha *et al*, 1989). Cells were incubated with a range of indomethacin concentrations for 4 days at 37°C and 5% CO_2_ in a humidified environment. The effect of si-MRP1 downregulation on cell survival was tested in the MTT assay using continuous incubation with indomethacin. After a 4-day culture period, MTT (5 mg ml^−1^ in PBS) was added and formazan crystal production was measured as described previously. Controls consisted of media without cells (background extinction) and cells incubated with medium instead of the cytotoxic agent. Experiments were performed three times in quadruplicate.

### Apoptosis assay

The effect of GSH on indomethacin-mediated apoptosis induction was tested in cells (1.5 × 10^4^ well^−1^) cultured in 96-wells plates and preincubated with 250 *μ*M BSO for 24 h. Apoptosis was induced by adding different concentrations of indomethacin for 24 h.

In addition, GLC_4_-Adr cells were transfected with si-MRP1 and si-Luciferase. After 48 h these cells were plated in 96-wells plates and exposed to different concentrations of indomethacin for 24 h. Acridine orange staining (10 *μ*g ml^−1^) was used to identify apoptotic cells. Apoptosis was defined as the appearance of apoptotic bodies and/or chromatin condensation, viewed using a fluorescence microscope. Results were expressed as the percentage of apoptotic cells in a culture by counting at least 200 cells per well. All apoptosis assays were performed in duplicate and repeated three times.

### Glutathione assay

Free GSH was measured by a modified method as described by Allen *et al* (2000). Cells were plated in 25-cm^2^ flasks and exposed to 250 *μ*M BSO for 24 h to achieve adequate GSH depletion. Cells were exposed to indomethacin and 50 *μ*M MK571 for 2 h to evaluate short-term effect of these drugs on GSH levels. Cells were isolated from six-well plates and washed in PBS. Protein was precipitated with 5% TCA and the precipitate was spun down at 4500 **g**. The supernatant was diluted to 1 mg ml^−1^ protein. A 150-*μ*l volume of the supernatant was put in a 96-well plate in different dilutions. Ellman's reagents (12 mM DTNB, 5 mM EDTA in 125 mM phosphate buffer) and 10 *μ*l of 125 mM phosphate buffer containing 5 mM EDTA and 0.4 U of glutathione reductase were added to the samples. The reaction was started by the addition of 20 *μ*l 5.6 mM NADPH in 125 mM phosphate buffer containing 5 mM EDTA. 5,5′-Dithio-bis(2-nitrobenzoic acid reduction produced in the assay was measured at 405 nm. All GSH assays were performed three times.

### Mitochondrial membrane potential

To determine whether there is an absolute decrease in fluorescence, 3 × 10^5^ cells were seeded in six-well plates and exposed to different concentrations of indomethacin for 24 h. MitoTracker red® (300 nM)was added for 45 min at 37°C and cells were analysed with an Elite flow cytometer (Bechman Coulter, Fullerton, CA, USA). MitoTracker red fluorescence was measured in the PE channel. Experiments were performed three times and a representative example was shown.

### Statistical analysis

Paired Student's *t*-test, independent samples *t*-test or one-sample *t*-test was used to calculate statistical differences. Differences were considered significant when *P*<0.05.

## RESULTS

### The effect of MRP1 downregulation on indomethacin-induced apoptosis and cell death

Compared with the parental cell line GLC_4_, the doxorubicin-resistant sub-line GLC_4_-Adr strongly overexpresses MRP1 and is highly sensitive to indomethacin (De Groot *et al*, 2005). The role of MRP1 in indomethacin sensitivity was investigated using an MRP1 siRNA approach. Efficient downregulation of MRP1 mRNA was demonstrated, since the relative MRP1 mRNA expression levels, as determined with quantitative RT–PCR, were reduced to 55.2±18.3% in si-MRP1-transfected GLC_4_-Adr cells, and to 44.9±1.1% in si-MRP1-transfected GLC_4_ compared with their respective si-Luciferase-transfected controls. The absolute MRP1 mRNA expression levels, however, were 79 times higher in GLC_4_-Adr compared with GLC_4_ cells.

**Figure 1:**
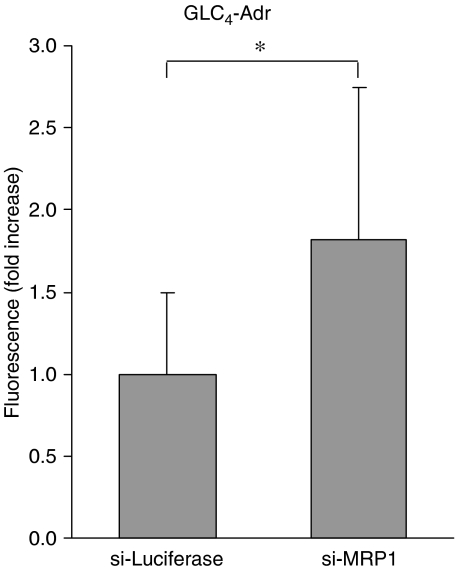
Relative cellular CF fluorescence after si-MRP1 and si-Luciferase treatment (^*^P<0.05).

The CF accumulation following 1 h CFDA exposure was used to determine the MRP1 function in siRNA-transfected GLC_4_-Adr cells. Carboxyfluorescein accumulation was 84±19% higher in the si-MRP1-transfected cells compared with the si-Luciferase-transfected cells, indicating that the MRP1 function in GLC_4_-Adr was indeed decreased as a result of MRP1 mRNA downregulation (Figure 1).

**Figure 2:**
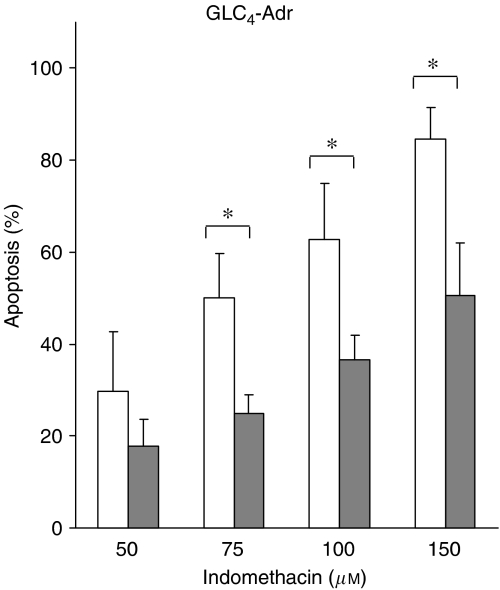
Indomethacin-induced apoptosis in GLC_4_-Adr 48 h after si-Luciferase transfection (white) or si-MRP1 transfection (grey). Cells were exposed to indomethacin for 24 h. No apoptosis was observed in GLC_4_ (data not shown). Data represent the mean±s.d. of three independent experiments (^*^P<0.05).

Indomethacin-induced apoptosis was clearly reduced in si-MRP1-transfected GLC_4_-Adr cells (Figure 2). For example, a reduction in apoptosis (2.0±0.05-fold) was observed at 75 *μ*M indomethacin. At higher indomethacin concentrations, similar reductions were observed in si-MRP1-transfected GLC_4_-Adr cells compared with si-Luciferase-transfected GLC_4_-Adr cells. However, indomethacin concentrations up to 150 *μ*M did not induce apoptosis in either si-MRP1- or si-Luciferase-transfected GLC_4_ cells (data not shown).

**Figure 3:**
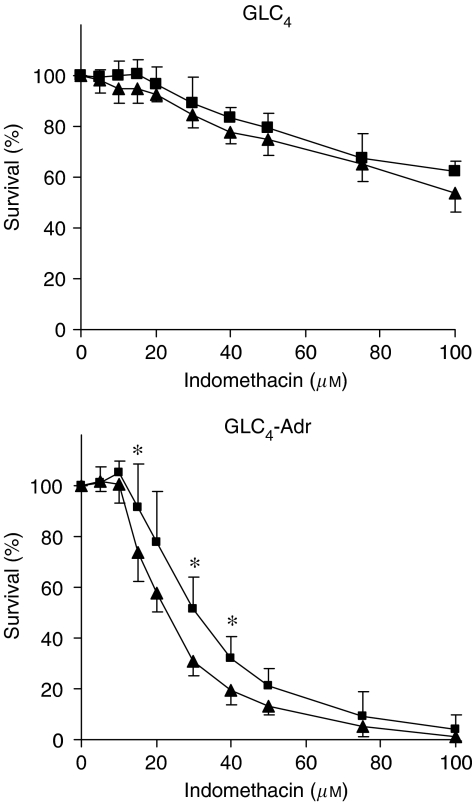
Indomethacin-induced growth inhibition. Survival of GLC_4_ and GLC_4_-Adr cells 48 h after si-Luciferase (triangle) or si-MRP1 (square) transfection was compared. Data represent the mean±s.d. of three independent experiments (^*^P<0.05).

Apart from a reduction in apoptosis, an increased survival following indomethacin exposure was also observed in si-MRP1-transfected GLC_4_-Adr cells (Figure 3). The IC_50_ for indomethacin was 22.8±2.6 *μ*M in si-Luciferase-transfected GLC_4_-Adr and 30.4±5.1 *μ*M in si-MRP1-transfected GLC_4_-Adr (*P*<0.05). No differential effect on cell survival was observed between GLC_4_ cells transfected with si-Luciferase or si-MRP1 for indomethacin concentrations up to 100 *μ*M.

### Effect of indomethacin on GSH levels

To investigate whether the protective effect of MRP1 siRNA against indomethacin-induced apoptosis is not only related to a reduction in MRP1 activity but also to an effect on cellular GSH levels, we measured intracellular GSH concentrations. GLC_4_ cells have a slightly lower although not significant GSH content of 9.9±2.2 *μ*g mg of total cellular protein compared with GLC_4_-Adr cells with a cellular GSH content of 11.1±6.1 *μ*g mg^−1^ (*P*=0.75).

Interfering with the MRP1 function did not affect cellular GSH levels in either cell line. First, downregulation of MRP1 with si-MRP1 transfection did not alter intracellular GSH content in GLC_4_ and GLC_4_-Adr. Second, when cells were exposed to 50 *μ*M of the specific MRP1 inhibitor MK571 for 2 h, GSH levels were not affected and no apoptosis was induced. Higher concentrations of MK571 up to 100 *μ*M did induce apoptosis (data not shown). Indomethacin exposure alone, however, lowered GSH levels by 31.5±19.8% in GLC_4_-Adr cells (*P*<0.05) and by 20.6±37.7% in GLC_4_ cells (*P*=0.28) (Figure 3).

**Figure 4:**
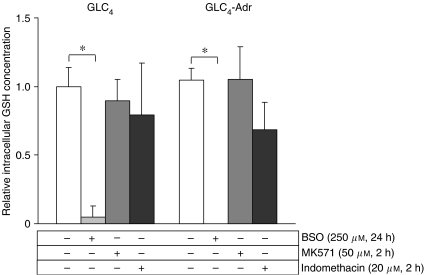
Relative GSH levels in GLC_4_ and GLC_4_-Adr cells. Glutathione intracellular concentration was determined after 24 h BSO, 2 h MK571 or 2 h indomethacin exposure. Glutathione levels were calculated as the relative concentration compared with untreated cells. Data represent the mean±s.d. of three independent experiments (^*^P<0.05).

**Figure 5:**
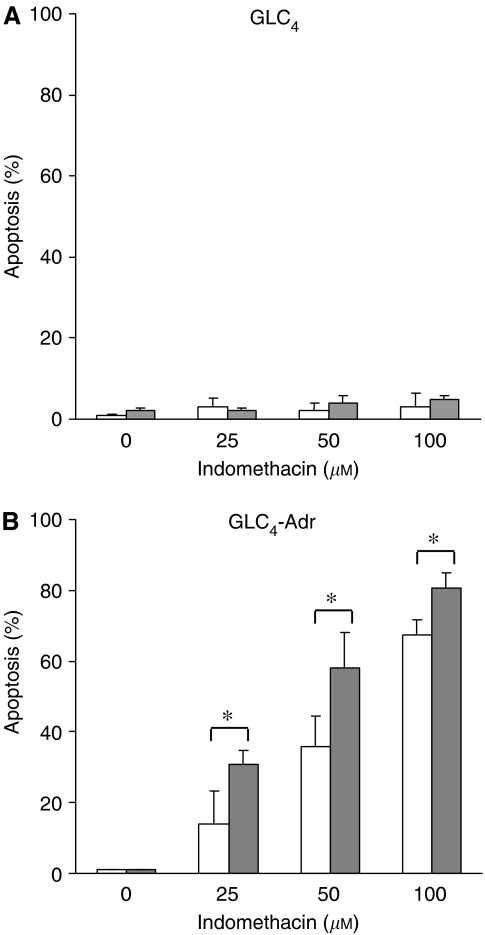
Indomethacin-induced apoptosis in GLC_4_ (**A**) and GLC_4_-Adr (**B**) after 24 h BSO exposure (grey), or control (white). Cells were exposed to indomethacin for 24 h. No apoptosis was observed in GLC_4_. Data represent the mean±s.d. of three independent experiments (^*^P<0.05).

To confirm the relationship between GSH levels and indomethacin-induced apoptosis, cellular GSH levels in both cell lines were reduced with BSO treatment. As shown in Figure 4, GSH levels were diminished after BSO treatment in GLC_4_-Adr cells. This resulted in an increase in apoptosis levels in GLC_4_-Adr cells treated with indomethacin in combination with BSO compared with GLC_4_-Adr cells treated with indomethacin only, for example, from 14.1 to 35.8% apoptosis at 25 *μ*M indomethacin (Figure 5). However, at higher indomethacin concentrations, the relative increase in apoptosis was less pronounced. Indomethacin in combination with BSO did not induce apoptosis in GLC_4_ at any of these concentrations.

These results indicate that cellular GSH levels are not primary determinants for MRP1-independent indomethacin-induced apoptosis, since GSH depletion did not result in indomethacin-induced apoptosis in GLC_4_ cells. Furthermore, it suggests that at relatively low indomethacin concentrations GSH levels may be determinants of indomethacin-induced apoptosis in an MRP1-dependent manner.

### Indomethacin-induced loss of mitochondrial membrane potential

**Figure 6:**
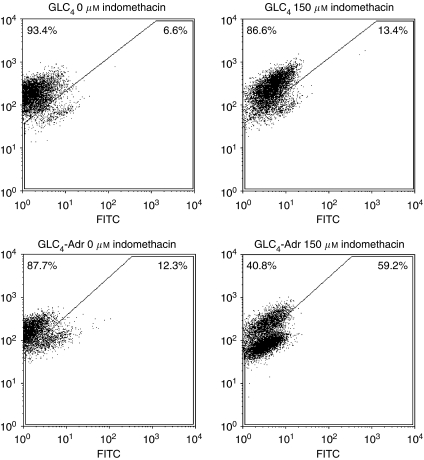
Flow cytometric analysis of intact mitochondria in GLC_4_ and GLC_4_-Adr cells after 24 h exposure to indomethacin and stained with MitoTracker red. Cells not exposed to indomethacin were compared with cells exposed to 150 *μ*M indomethacin. A representative example is shown.

Previously, we have demonstrated that indomethacin-induced apoptosis in GLC_4_-Adr was caused by caspase-8 as well as caspase-9 activation (De Groot *et al*, 2005). However, the role of mitochondria in indomethacin-induced apoptosis in GLC_4_-Adr was not established. Therefore, we determined whether indomethacin-mediated apoptosis induction coincided with loss of mitochondrial membrane potential in the cells. Flow cytometry revealed that after indomethacin exposure for 24 h two GLC_4_-Adr cell populations exist, a MitoTracker red-high and a MitoTracker red-low population (Figure 6). The presence of the MitoTracker red-low population (40.8% at 150 *μ*M of indomethacin) at 24 h suggests that loss of mitochondrial membrane potential in GLC_4_-Adr occurs in conjunction with apoptosis induction, as at 100 *μ*M indomethacin, up to 84.6% of apoptosis was induced. Indomethacin had no effect on mitochondrial membrane potential in GLC_4_ (Figure 6).

## DISCUSSION

This is the first study demonstrating that indomethacin can induce apoptosis via an MRP1-dependent mechanism in the MRP1-overexpressing SCLC cell line GLC_4_-Adr. Efficient functional downregulation of MRP1, as shown using the elevated cellular level of the MRP1 substrate CF, resulted in less apoptosis and concomitantly in enhanced survival of GLC_4_-Adr cells following indomethacin exposure. In contrast, MRP1 downregulation in GLC_4_ cells had no effect on indomethacin-induced apoptosis or survival. Glutathione levels in GLC_4_-Adr cells were decreased following indomethacin exposure, but these levels were not affected by MRP1 downregulation. In addition, depletion of cellular GSH by BSO made GLC_4_-Adr cells more sensitive to indomethacin. Since GSH depletion by BSO did not enhance indomethacin-induced apoptosis in GLC_4_, our results suggest that indomethacin induces apoptosis via an MRP1-dependent mechanism, which may partially be dependent on cellular GSH levels. Another MRP1-dependent mechanism involved is the indomethacin-induced activation of the mitochondrial apoptosis pathway, which is reflected in loss of the mitochondrial membrane potential simultaneously with apoptosis induction in GLC_4_-Adr. These results confirm our previous finding that indomethacin induces apoptosis via activation of caspase-8 as well as caspase-9 in GLC_4_-Adr (De Groot *et al*, 2005).

Indomethacin is a well-known inhibitor of glutathione-*S*-transferase and cyclooxygenase, but it can also affect MRP1 (Draper *et al*, 1997; Roller *et al*, 1999). Indomethacin increases chemotherapy sensitivity through inhibition of MRP1 function and stimulation of the GSH efflux in MRP1-overexpressing cell lines, as demonstrated in polarized Madin–Darby canine kidney (MDCKII) cells (Evers *et al*, 2000). Drug-induced GSH efflux through MRP1 has been described for verapamil in baby hamster kidney-21 cells transfected with human MRP1 (Trompier *et al*, 2004). The GSH levels in GLC_4_ and GLC_4_-Adr are similar, but GSH turnover is much higher in GLC_4_-Adr due to its increased MRP1 expression (Kool *et al*, 1997). In the present study, we demonstrated that GSH levels in GLC_4_-Adr did not rise after 2 h exposure to indomethacin. In contrast, GSH levels were even slightly decreased in GLC_4_-Adr but not in GLC_4_, suggesting that indomethacin-associated MRP1 inhibition does not cause indomethacin-induced apoptosis.

NSAIDs including indomethacin can be metabolized intracellularly generating reactive oxygen species (ROS). The formation of ROS has actually been implicated in indomethacin idiosyncratic toxicity such as bone marrow toxicity or hepatitis (Galati *et al*, 2002). ROS are inactivated by mitochondrial GSH. In our model, cellular GSH levels were decreased by short exposure to indomethacin. Moreover, the reduction of GSH levels by BSO, a drug that specifically inhibits GSH production, increases indomethacin-mediated apoptosis in GLC_4_-Adr but not in GLC_4_. A reduction in cellular GSH level can be an important trigger for apoptosis induction as demonstrated in the B-cell lymphoma cell line PW after exposure to BSO (Armstrong *et al*, 2002). Cellular GSH depletion also resulted in apoptosis in a caspase-8-dependent manner in NCI-H889 SCLC cells, following treatment with the proteasome inhibitor MG132 (Bang *et al*, 2004). In addition, mitochondria of GSH-depleted cells can become more sensitive to oxidative stress (Henry-Mowatt *et al*, 2004). Oxidative stress then resulted in the loss of mitochondrial membrane potential and the release of several essential players of the mitochondrial apoptosis pathway, such as cytochrome *c* and apoptosis-inducing factor into the cytosol as well as the activation of caspases and apoptotic protease-activating factor-1 (Kannan and Jain, 2000; Henry-Mowatt *et al*, 2004). Previously, we have shown that inhibition of active caspase-9 decreased indomethacin-induced apoptosis in GLC_4_-Adr cells by 44%, suggesting the involvement of the mitochondrial apoptosis pathway (De Groot *et al*, 2005). In the present study, we demonstrated that indomethacin actually causes loss of mitochondrial membrane potential and subsequently results in the activation of the mitochondrial apoptosis pathway in GLC_4_-Adr cells. Further research is needed to establish the role of oxidative stress in the loss of the mitochondrial potential. Taken together our results indicate that indomethacin-induced apoptosis in GLC_4_-Adr cells is dependent on MRP1 expression. Indomethacin-induced apoptosis is accompanied by loss of mitochondrial membrane potential. Activation of this pathway may be further enhanced by caspase-8 activation and Bid cleavage, which we have reported earlier for indomethacin-treated GLC_4_-Adr cells (De Groot *et al*, 2005).

Downregulation of MRP1 and concomitant loss of MRP1 function slightly decreased indomethacin sensitivity indicating that MRP1 activity facilitates indomethacin-mediated apoptosis. However, one has to take in account that the MRP1 downregulation was not complete, so the effect of MRP1 activity on indomethacin-induced apoptosis is probably underestimated in this model. In addition, GSH depletion by BSO increases indomethacin-mediated apoptosis in GLC_4_-Adr only. These two findings indicate that the MRP1 function is important in indomethacin-induced apoptosis. The exploitation of a chemotherapy resistance factor such as MRP1 to induce apoptosis is a novel and potentially interesting finding.

In the future, identification of MRP1-overexpressing tumours and exposure of these tumours to combination therapies including indomethacin may provide a novel approach in the treatment of MRP1-overexpressing cancers.
